# First Come, First Served: Sui Generis Features of the First Intron

**DOI:** 10.3390/plants9070911

**Published:** 2020-07-19

**Authors:** David Zalabák, Yoshihisa Ikeda

**Affiliations:** Department of Molecular Biology, Centre of the Region Haná for Biotechnological and Agricultural Research, Faculty of Science, Palacký University, 78371 Olomouc, Czech Republic; david.zalabak@upol.cz

**Keywords:** first intron, alternative splicing, intron-mediated enhancement, upstream ORF

## Abstract

Most of the transcribed genes in eukaryotic cells are interrupted by intervening sequences called introns that are co-transcriptionally removed from nascent messenger RNA through the process of splicing. In *Arabidopsis*, 79% of genes contain introns and more than 60% of intron-containing genes undergo alternative splicing (AS), which ostensibly is considered to increase protein diversity as one of the intrinsic mechanisms for fitness to the varying environment or the internal developmental program. In addition, recent findings have prevailed in terms of overlooked intron functions. Here, we review recent progress in the underlying mechanisms of intron function, in particular by focusing on unique features of the first intron that is located in close proximity to the transcription start site. The distinct deposition of epigenetic marks and nucleosome density on the first intronic DNA sequence, the impact of the first intron on determining the transcription start site and elongation of its own expression (called intron-mediated enhancement, IME), translation control in 5′-UTR, and the new mechanism of the *trans*-acting function of the first intron in regulating gene expression at the post-transcriptional level are summarized.

## 1. Introduction

More than four decades ago, we underwent a paradigm shift in gene organization in higher organisms and caught a glimpse of the molecular processes in manufacturing messenger RNA from heterogeneous nuclear RNA (hnRNA). In 1977, electron microscopy images visualizing DNA:RNA hybrids annealed between adenovirus 2 hexon mRNA and restriction endonuclease cleavage fragments of viral DNA were the smoking gun proof demonstrating that cytosolic mRNA is matured through the removal of intervening sequences from nascent mRNA, called splicing [1,2]. In the following year, 1978, the terms “introns” and “exons” were proposed for the first time to describe a mosaic feature of eukaryotic genes, comprising coding sequences (exons) interrupted by intragenic regions (introns), by Gilbert, who wondered “Why genes in pieces?” [3]. He also coined the statement “Evolution can seek new solutions without destroying the old” [3]. Instead of duplicating the genetic materials, higher organisms take advantage of alternative splicing (AS) to increase protein diversification. Despite the fact that more than 40 years have passed since its discovery, very simple questions such as the origin and evolution of spliceosomal introns remain unsolved [4] and have been contentious in the intensive, long-standing “introns-early/introns-late” debate [5,6]. Either way, although intron densities among eukaryotes are variable, introns and spliceosome machinery are ubiquitous in eukaryotes [7,8] Regulatory molecular mechanisms of pre-mRNA processing [9], benefits of splicing that contribute to the diversification of proteins by AS [10], epigenetic regulation of AS [11], the roles of introns in modulating expression in diverse regulatory layers [9], how introns influence and enhance eukaryotic gene expression. [9,12], and AS in plant development and stress responses [13,14,15,16] have been intensively discussed and summarized elsewhere. Along with providing concise and general background knowledge of AS, we will focus particularly on unique signatures of the first intron sequence on DNA/nucleosome and on pre-mRNA in its own gene regulation. Additionally, we will highlight the novel functions of yeast first introns modulating the translation of other genes for fitness to nutrient deprivation.

## 2. First Intronic DNA Sequence

In humans, the first intronic DNA sequence is the longest and most conserved, and exhibits the highest density of regulatory chromatin marks when compared to the remaining intronic sequences [17,18]. DNA and histone methylation levels are also related to nucleosomes [14,15,16]. In general, the DNA methylation level correlates with that of H3K4me1 but is exclusive with H3K4me2 and H3K4me3 in transcribed genes [19]. Similar to animal chromosome organization [20], genome-wide nucleosome positioning analysis in *Arabidopsis* revealed that both 5′- and 3′-splice sites (5′-SSs and 3′-SSs) are preferentially enriched in nucleosomes, nucleosomal DNA is more highly methylated, and nucleosomes are enriched in exons [21]. Interestingly, nucleosome deposition at the first intron 3′-SS is more clearly defined than the other intron 3′-SSs [22]. Repressive epigenetic marks such as DNA methylation and histone di-methylations are deposited on some introns, especially on long introns (>2 kb) [23]. In human cells, histone modifications such as H3K4me3 and H3K9ac are enriched in the 5′-SS of the first intron [24]. Recent findings suggest an effect of DNA methylation on splicing [25,26] In humans, DNA methylation is known to affect 22% of AS, presumably through (1) modulation of the elongation rate of RNA polymerase II, or (2) the formation of protein bridges of HP1 that recruit splicing factors onto exons [26,27] IR in plants is highly enriched in DNase I hypersensitive sites (DHSs) [28]. In rice, approximately 7% of AS events in *met1-2* null allele, whose methyl-CG level is globally lost, are affected, suggesting the involvement of DNA methylation in AS, but the contribution is relatively minor [29].

When intronic regions are larger, regulatory elements are likely to be embedded within them to regulate their gene expression [30,31] A well-documented example of this is intronic transposable elements (TEs) [32]. Around 3% of TEs are located within gene bodies, with the majority at intronic regions [33] often triggering the formation of transcriptionally repressive heterochromatin [32]. The second example of an intron-encoded element is microRNA (miRNA). Similar to canonical intron-containing gene expression, intron-embedded miRNA is co-transcribed with its host gene, and its subsequent maturation is controlled by AS. This regulation is unique and intriguing, in that environmental cues such as heat stress affect AS events to control the level of mature miRNA, which, in turn, regulates the miRNA-target gene expression *in trans* [34]. On the contrary, some intronic regions contain a sequence complementary to miRNA [35] or are targeted by noncoding RNA (ncRNA). HIDDEN TREASURE 1 (*HID1*) encodes ncRNA that forms a protein complex in the nucleus to bind to the *PIF3* first intronic sequence to repress *PIF3* expression at the transcriptional level [36]. This was probably the first case in plants demonstrating the regulation of ncRNA binding to chromatin for regulation of gene expression.

The FLOWERING LOCUS C (*FLC*) first intron contains two RY (R, purine; Y, pyrimidine) cis-elements (TGCATG) that are recognized by the B3 DNA binding domain of VAL1 (VIVIPAROUS1/ABI3-LIKE factor 1), a transcriptional repressor capable of repressing *FLC* expression by modifying the H3K27me3 level in the *FLC* intronic region and decreasing H3 acetylation in the first exon by recruiting plant homeodomain-polycomb repressive complex 2 (PHD-PRC2) [37].

## 3. Splicing is Predominantly Co-Transcriptional

**Figure 1:**
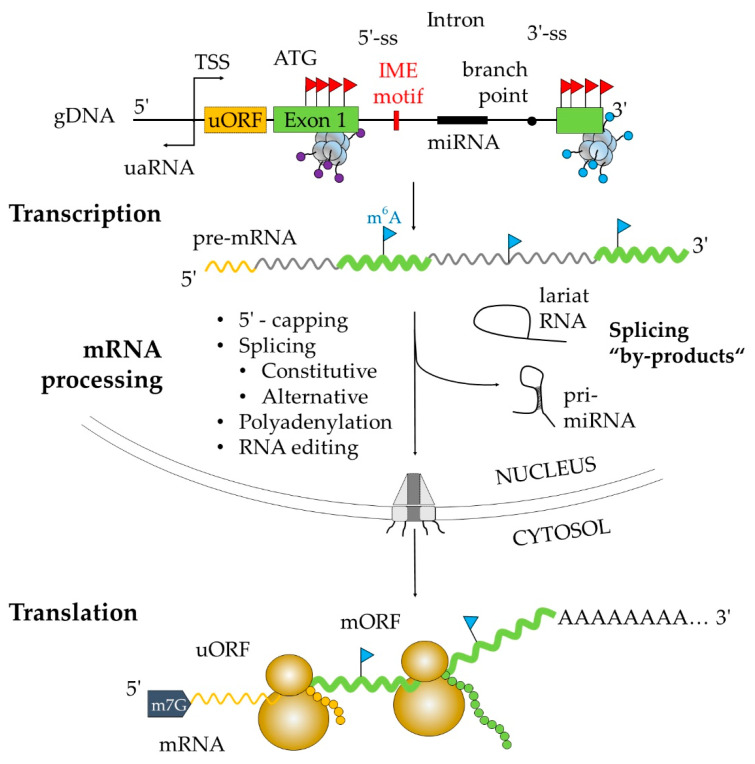
Organization of eukaryotic gene and key steps in gene expression. Structure of eukaryotic gene encoded in nuclear genomic DNA (gDNA) is shown on top. Orange box represents an upstream open reading frame (uORF); green boxes are exons of main open reading frame (mORF) carrying initiation codon (ATG). The first intron is flanked by a 5′-splice site (5′-SS) and a 3′-splice site (3′-SS), with branch point (black circle) located close to 3′-SS. The first intron might carry an intron-derived motif (IDM), gene encoding micro RNA (miRNA) or miRNA-binding site. Transcription is initiated from transcription start site (TSS) in sense orientation, producing premature messenger RNA (pre-mRNA) and, to a lesser extent in antisense orientation, upstream antisense RNA (uaRNA). Gene transcription is further regulated by specific epigenetic marks, e.g., CpG methylation (red flags) enriched in exonic regions of gDNA and adenine methylation (blue flags) within RNA molecule. Another layer of epigenetic control is mediated by histone (grey octamer structure) methylation as H3K4me2 together with H3K4me3 pattern (purple dots) are enriched in the 5′-end and H3K4me1 (blue dots) in the 3′-end of gene body. pre-mRNA is further processed to mature messenger RNA (mRNA) and exported to cytosol through nuclear pores. The processing involves 5′-capping (m7G), splicing, polyadenylation, and occasionally RNA editing. During the splicing process, lariat RNA is produced. Primary miRNA (pri-miRNA) is released from spliced intron-containing miRNAs and requires additional processing to become functional. Translation of mRNA into protein occurs on ribosomes composed of small and large subunits (yellow structure).

As described above, exonic and intronic DNA exhibit distinct nucleosome occupancy/chromatin structure, as well as DNA and histone depositions, all of which could define RNA polymerase II (Pol II) elongation speed. Due to high GC content and concentrated nucleosomes in the exonic region, elongation speed is slower than that in the intronic region (Figure 1, top) [38,39] As a result, AS is regulated by the speed of transcriptional machinery [11,40,41,42,43] A myriad of articles demonstrate that splicing in many cases takes place co-transcriptionally [39,44,45,46] and the speed of transcription elongation driven by Pol II affects splicing [40,41,47] In the case of plants, the rate of splicing appears to be much slower than that of yeast. Jia et al. showed that by developing a nanopore-based method, more than half of the introns remain unspliced after Pol-II transcribed 1 kb past the 3′-splice site, and the authors called such introns post-transcriptionally spliced introns (pts introns) [48].

Throughout the whole processes of transcription, Pol II interacts with various proteins (such as transcription factors, chromatin modifiers, and RNA processing enzymes) via its carboxy-terminal domain (Pol II-CTD) in post-translational modification, especially in a phosphorylation-dependent manner [49]. It is worth noting that Pol II interacts with active spliceosome complex (both snRNA and proteins) in a Ser-5 phosphorylation-dependent manner [46]. The distribution of Ser-5P Pol II has a particular peak at the 5′-SS of exon–intron boundaries, and Ser-5P Pol II accumulation persists over exon sequences; however, such accumulation is diminished at the 5′-SS of exon excluded by exon skipping. This finding suggests that Ser-5P Pol II pauses at 5′-SSs to allow time for the U2 snRNP splicing complex to cleave 5′-SS [33].

Nuclear cap-binding complex (CBC) consists of two subunits, CBP20 and CBP80, which bind to the cap structure of transcripts and affect splicing [50]. From the 5′-end of pre-mRNA, the CBC interacts with U1 snRNP at the 5′-SS of the first intron [50]. In *Arabidopsis*, cap-binding proteins, CBP20 and CBP80, and its interacting partner SERRATE (*SE*) modulate the first intron AS by affecting the 5′-SS of the first intron [51,52,53]

**Figure 2:**
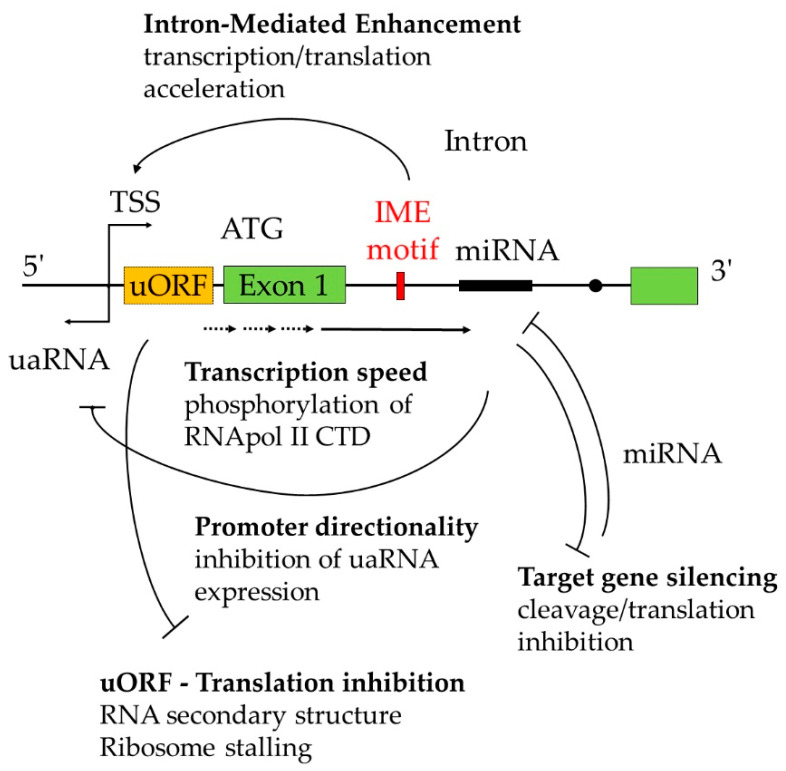
The first intron plays a key role in several mechanisms regulating gene expression. The presence of intron-mediated enhancement (IME) motif within the first intron determines the TSS and increases gene expression. Phosphorylation status of polymerase II (Pol II) carboxy-terminal domain (CTD) varies during transcription. Ser-5P Pol II poses at the exon–intron junction. The first intron inhibits transcription of uaRNA by recruiting termination factors that confer promoter directionality. The presence of uORF within the mRNA transcript might negatively affect the translation efficiency of main ORF by forming the secondary structure or by ribosome stalling. Another layer of gene expression control is mediated through miRNA/TE encoded within intronic DNA. Alternatively, the intronic sequence is complementary to miRNA.

Non-protein-coding RNAs are shown to modulate AS, especially IR in plants. AS COMPETITOR LONG NONCODING RNA (*ASCO*-lncRNA) highjacks AS regulators by interacting with NUCLEAR SPECKLE RNA-BINDING PROTEIN (*NSR*), resulting in IR of NSR-target transcripts [54]. In many cases, micro RNA (miRNA) molecules are complementary to mRNA sequences; however, some miRNAs found in *Arabidopsis* and rice genomes target intronic sequences [35], raising the possibility that miRNA regulates pre-mRNA (Figure 2). Meng et al. [35] proposed a novel regulatory cascade called “miRNA-intron-phased secondary sRNAs targets” by which such overlooked annotated intron-targeting miRNAs are processed by ARGONATURE 1 silencing complex to form mature miRNAs that bind to and cleave target pre-mRNAs in the nucleus. As a consequence, cleaved remnants are subjected to the RNA-dependent RNA polymerase template to give rise to the production of sRNA by DICER to degrade target mRNA in the cytoplasm or modify target intronic DNA sequences (such as methylation) as AGO4-associated sRNA in the nucleus (experimental validation is required) [35]. miRNAs complementary to the first intronic DNA are listed in Table 1.

## 4. Intron-Mediated Enhancement (IME), a Mysterious Phenomenon

It is widely recognized that the presence of intron stimulates mRNA accumulation [12]. Back in 1979, two years after the discovery of intervening DNA in the adenovirus 2 genome [1,2] it was reported that intron was required for the accumulation of stable mRNA [12]. In 1987, the same outcome was confirmed by studying ALCOHOL DEHYDROGENASE 1 (*ADH1*) in maize [55], and the authors revealed that only the first intron is sufficient to retain expression similar to that of the genomic *ADH1* construct [55,56] This result suggests a unique ability of the first intron to enhance its expression, yet the precise molecular mechanism underlying intron-mediated enhancement (IME) remains vague (Figure 2).

### 4.1. Splicing-Dependent IME

The majority of mammalian promoters initiate transcription on both sides in opposite directions, called divergent transcription [57,58,59] whereas plant promoters appear to act unidirectionally [44,60] One way to increase mRNA accumulation is to orient transcription initiation toward one direction. The splicing signal has been shown to play a predominant role in enhancing gene transcription [61]. This was corroborated in yeast cells, showing that splicing-competent introns affect promoter directionality, favored toward transcribing downstream coding region producing mRNA rather than the upstream antisense RNA [62]. The mechanism behind to account for the splicing-competent IME appears to be gene looping. Gene loops juxtapose promoters and terminators in yeast [63,64]. O’Sullivan et al. showed that a looped configuration of genes (where promoter and terminator regions are in close proximity by physical interaction) is associated with early transcriptional activation [63]. It appears that the looped configuration is indispensable for splicing-competent intronic DNA to enhance mRNA accumulation and that the gene looping is crucial for the recruitment of termination factors in the promoter proximal region of an intron-containing gene [62]. Due to the unidirectional nature of plant promoters, it is not entirely clear whether or not IME in plants is achieved in a splicing-competent intron-dependent manner.

### 4.2. Splicing-Independent IME in Plants

Investigations into plant IME have been extensively carried out by Rose’s group [65,66]. For instance, first introns of maize *ADH1* [55], SHRUNKEN-1 [67,68] *GAP1* [69], *UBI1* [70], rice *SODCC2* (*cytosolic* superoxide dismutase) [71], *Arabidopsis PAT1* (phosphoribosylanthranilate transferase 1) [72], UBIQUITIN (*UBQ*) genes [73], and *MHX* [74,75] have been documented to boost mRNA accumulation. Hereafter, intron that can significantly increase gene expression is termed “stimulating intron.” Based on the assumption that introns in close proximity to TSS would be more frequently enriched in IME signals than distal introns, genome-wide in silico surveys of *Arabidopsis* and rice were carried out, and a word-based discriminator called the IMEter was developed [76]. Consequently, the most common motifs found using NestedMICA were CGATT and TTNGATYTG in *Arabidopsis* and TCGATC in rice [76,77,78]. Hereafter, the TTNGATYTG sequence is termed “IME motif.” A similar consensus sequence responsible for IME in rice was found too: GATCTG [71]. The ability of IME motif enriched in promoter-proximal introns to boost mRNA accumulation was confirmed by a gain-of-function approach of converting non-stimulating intron into stimulating intron by integrating IME motif [77]. The unique features of the IME motif found in *Arabidopsis* have been further revealed. Contrary to IME observed in yeast cells [61,62], splicing is not required for plant-stimulating introns [79,80] The *UBQ10* first intron not only increases mRNA accumulation, but also affects tissue specificity when the reporter gene containing the *UBQ10* first intron is under the control of different promoters [81], suggesting that *UBQ10* intron renders tissue specificity over promoter sequence. Likewise, the first intron of PROFILIN2 (*PRF2*), encoding a small actin-binding protein expressed in vegetative organs, is capable of boosting mRNA accumulation and confers vegetative tissue expression when driven by reproductive tissue-specific *PRF5* promoter [82].

Intriguingly, the *UBQ10* first intron determines the transcription start site (TSS) from where it locates (Figure 2) [83]. In order for an intron to accumulate mRNA, its position has to be within 1000 bp downstream from the TSS [83,84]. Contrary to its name (intron-mediated enhancement), IME takes place in a splicing-independent manner because its ability to boost mRNA accumulation persists even when IME motif is inserted in 5′-UTR (IMEter score is high in 5′-UTR) or present in the coding region (as long as it is positioned within 1000 bp downstream from TSS) [85,86]. Although IME motif is capable of increasing translational efficiency [72,75], it is likely that it is more relevant to IME when located in the DNA rather than the strand-specific nascent RNA (prior to splicing) [87], which is in agreement with its ability to determine TSS [83].

## 5. Post-Transcriptional Regulation by Intron Retention (IR) within 5′-UTR

Intron retention (IR) is the major AS in plants [88,89], but the idea that IR contributes to protein diversity is controversial [90,91,92]. The outcome of IR differs depending on the position of the retained first intron, or most AS events are the consequence of stochastic noise in the process machinery [93]. In plants, around 15% of all AS events take place within the 5′-UTR, which can affect the transport and stability of mRNAs that are kept under constant surveillance by the nonsense-mediated decay (NMD) pathway [10,94,95].

When AS of the first intron within 5′-UTR takes place, the impact on gene expression will be quite the opposite. A positive effect of the first intron retention on boosting mRNA accumulation was shown by Laxa et al.: the first intron within 5′-UTR of Glu:glyoxylate aminotransferase 1 (*GGT1*) contains a TGTGATTTG sequence that is highly similar to IME motif and enhances leaf-specific expression at the transcriptional level by enriching Pol II abundance [96]. The topological secondary structure built by base-pairing also accounts for the accessibility of translational machinery. First intron retention within 5′-UTR of *ZIF2* (encoding ZINC-INDUCED FACILITATOR 2) is shown to provide a stable stem-loop structure that enables more efficient translation when compared to the spliced variant, in which the stable structure is lost by splicing [97]. On the contrary, base-pairing interaction between retained intron and 5′-UTR of yeast *HAC1* represses translation [98].

The vast number of eukaryotic mRNAs contain a short open-reading frame within their 5′-UTR, called upstream ORF (uORF). In *Arabidopsis*, about 35% of genes transcribing mRNA contain at least one uORF by prediction [99], and high-resolution ribosome profiling verified 187 uORFs being translated [100]. The presence of uORF within 5′-UTR negatively affects the translation of main ORF by ribosome stalling [101,102,103]. Although it is a process whose molecular mechanism is still being investigated, the repression of main ORF by uORF acts in both a uORF peptide translation-dependent [104] and -independent manner [105,106].

Riboswitches are regulatory elements in mRNA, predominantly found in the noncoding region, that act as metabolite sensors by selectively binding to ligands and regulate transcription or translation without the need for protein factors [107]. It is prevalent in prokaryotes, and the most widespread riboswitch known in bacteria responds to the coenzyme thiamine pyrophosphate (TPP), which is also conserved in fungi and plants [108,109,110]. Álvarez et al. proposed alluring retrograde signaling as a feedback regulatory mechanism of carotenogenesis, where *ASV1*, one of the splicing variants containing longer 5′-UTR of PHYTOENE SYNTHASE (*PSY*) mRNA, acting as a riboswitch-like module to repress translation and AS of the first intron is the key to modulating retrograde signaling [111]. Similarly, a phytohormone riboswitch has been proposed. Grojean and Downes hypothesized the involvement of riboswitch in cytokinin perception (beyond membrane-bound histidine kinase cytokinin receptors AHK2, AHK3, and AHK4) in *Arabidopsis* [112]. Having gone through biochemistry combined with bioinformatics data of the cytokinin-responsive gene, one candidate encoding CYSTEINE-RICH RECEPTOR-LIKE KINASE 10 (*CRK10*), whose expression is downregulated by cytokinin application, is likely to contain an adenine aptamer-related sequence in its first intronic sequence [112]. In both cases, further experimental results to corroborate their hypothesis are anticipated.

## 6. Regulation of First Intron Retention Located Downstream of ATG

IR that takes place in introns positioned after the ATG affects protein in its sequence, function, subcellular localization, or IR mRNAs is subjected to degradation by the NMD [113,114]. IR, in many cases, causes disruption of original ORF by premature stop codons that subsequently activate the NMD pathway. However, not all IR transcripts are sensitive to NMD [95]. The splicing of retained introns is facilitated by stress signals. Tomato WRKY transcriptional factor *SlyWRKY75* contains the first intron downstream of ATG, and its expression is regulated by *miR1127-3p*, which is complementary to the first intron sequence of *SlyWRKY75*. Under noninfected conditions, the first intron is retained, whereas upon *Botrytis cinerea* pathogen challenge, the overall *SlyWRKY75* transcript level is elevated, which is in sharp contrast to compromised *miR1127-3p* expression. Interestingly, pathogen challenges facilitate splicing of the retained first intron that remained unspliced under noninfected conditions [115]. These results imply that intron-targeting *miR1127-3p* binds to *SlyWRKY75* pre-mRNA in the nucleus to inhibit the splicing of target intronic region under noninfected conditions.

## 7. AS in Response to Stresses and Developmental Cues

Pathogen infection [115] is not the only example to show the effect of splicing a retained intron. Sessile plants have adopted sophisticated mechanisms to adjust to the varying environment, and the regulation of AS appears to play a more pivotal role than previously assumed. Splicing efficiency is regulated by stresses such as abiotic stress [116], drought [117], salt [118], cold [119,120], high temperature [34,121] phytochrome B signaling [122,123] thermo-priming [124], temperature-dependent regulation of circadian clock genes [125], and phosphate starvation [126]. Vernalization is, in part, regulated by AS of *FLC*, a central repressor in the flowering transition [37]. Tsugeki et al. suggested the crucial role of CLUMSY VEIN (*CUV*) encoding DEAD-Box RNA-dependent ATPase *PRP16* in facilitating the splicing of genes involved in auxin biosynthesis, transport, perception, and signal transduction [127]. AS of the splicing factor *At-RS31* has been shown to be regulated under retrograde signaling control [128]. Light/dark transition affects AS via the plastoquinone redox state [128], suggesting close communication between two organelles for fine-tuning of gene expression through AS. Such close communication is further supported by a study of LEFKOTHEA (*LEF*) encoding PORR domain-containing RNA-binding protein [129]. Nuclear-encoded LEF proteins exhibit dual targeting to the nucleus and chloroplasts to regulate AS of nuclear-encoded genes as well as group II introns in chloroplast [129]. According to the function and position of the first intron (relative to the main ORF ATG translation initiation site), genes whose expression is under the influence of AS of the first intron are summarized in Table 1. The biggest obstacle to account for the modulation of AS by environmental cues is the lack of knowledge of their perception mechanism (i.e., stress receptors). Besides, it should be noted that the spliceosome is among the largest molecular complexes in the cell, comprising five snRNAs and more than 150 proteins [130], which also impede our comprehension of stress-induced AS changes.

## 8. Intron Regulation beyond the Host Gene

In eukaryote cells, transcribing intronic regions and correctly finding junctions to cut and subsequently combine both ends are energy-consuming and laborious. Indeed, introns are junk on a good day but can be treasures on a bad day in the case of budding yeast [134]. In yeast, global splicing efficiency is affected by the expression level of ribosomal protein genes (RPGs) because the splicing apparatus is a limiting factor for non-RPG pre-mRNAs [135]. Parenteau et al. elegantly concluded that introns, as part of the unspliced pre-mRNA, promote resistance to nutrient starvation by enhancing the repression of RPGs [136]. In addition, a small subset of spliced introns with short distances between the lariat branch point and 3′-SS (lariat RNA, a byproduct of spliced introns) could also promote cell survival under starvation conditions [137]. Lariat RNAs control miRNA biogenesis by interacting with the dicing complex. A similar mechanism is likely to be conserved in humans [138] and plants [139]. *Arabidopsis* weak mutant allele, *dbr1-2*, defective in RNA debranching enzyme 1 (*DBR1*) accumulates lariat RNAs that act as a decoy by competing with the dicing complex to inhibit genome-wide miRNA processing [139]. Collectively, these results strongly suggest in trans regulation of excised intron beyond host gene expression.

## 9. Conclusions and Future Aspects

Information on defined epigenetic marks deposited on the first intronic DNA sequence and the higher sequence conservation within the longest first introns in all domains of life suggests that the first intronic DNA sequence is more distinguishable than the other introns. The underlying mechanisms of promoter-proximal introns to boost mRNA accumulation and determine TSS from downstream, a phenomenon called intron-mediated enhancement (IME), appear different from those of the initiation of DNA sequence-specific transcription by transcription factors or conventional enhancers. The biggest breakthrough for a better understanding of IME in plants was the discovery of the consensus IME motif enriched in introns capable of boosting mRNA accumulation [76]. However, it is not entirely clear whether the IME motif is unique in the plant kingdom.

When pondering the underlying mechanism for stimulating introns to determine TSS from the gene body in a relatively short range (up to about 1000 bp) [83], IME motif is likely to fulfill its potential when located in the DNA rather than when transcribed in pre-mRNA [87]. Is stimulating intron marked with specific epigenetic marks on DNA? Analogous to yeast, does stimulating intron facilitate a looped configuration of genes? What kind of underlying mechanisms are there for the enhancing intron to determine TSS? Our exploration of the unique features of plant first intron is ongoing.

## Figures and Tables

**Table 1:** Plant first intron with functions and stress responses.

Category	Gene Name (Gene ID)	Position Relative to ATG	Function	Organism	Reference
**IME**	*UBQ10* (At4g05320)	downstream	IME	*Arabidopsis*	[80]
	*PAT1/TRP1* (At5g17990)	downstream	IME	*Arabidopsis*	[72]
	*ADH1* (GRMZM2G442658)	downstream	IME	maize	[55]
	*Sh1* (GRMZM2G089713)	downstream	IME, splicing-dependent	maize	[56,68]
	*PRF1* (At2g19760)	downstream	IME	*Arabidopsis*	[82]
	*PRF2* (At4g29350)	downstream	IME	*Arabidopsis*	[82]
**miRNA/**	*OsCLV1* (Os1g07060)	upstream	target of miR2123abc	rice	[35]
**lncRNA**	*YSL3* (At5g53550)	upstream	miRNA-binding site	*Arabidopsis*	[35]
	*SLK2* (At5g62090)	upstream	miRNA-binding site	*Arabidopsis*	[35]
	*SlyWRKY75* (Solyc05g015850)	downstream	target of sly-miR1127	tomato	[115]
	n.a. (At1g32583)	upstream	source of miR400	*Arabidopsis*	[34]
	n.a. (At5g08185)	downstream	source of miR162a	*Arabidopsis*	[131]
	*NPF2.9/NRT1.9* (At1g18880)	downstream	source of miR837	*Arabidopsis*	[131]
	n.a. (At2g23348)	upstream	source of miR844	*Arabidopsis*	[131]
	*PIF3* (At1g09530)	upstream	target of HID1	*Arabidopsis*	[36]
**IR**					
**secondarystructure**	*ZIF2* (At2g48020)	upstream	translation enhancement	*Arabidopsis*	[97]
**riboswitch**	*PSY* (At5g17230)	upstream	translation inhibition	*Arabidopsis*	[111]
**riboswitch**	*CRK10* (At4g23180)	downstream	putative cytokinin-binding riboswitch	*Arabidopsis*	[112]
**uORF**	*PIF3* (At1g09530)	upstream	translation inhibition	*Arabidopsis*	[123]
**uORF**	*ARF3* (At2g24765)	upstream	translation inhibition	*Arabidopsis*	[132]
**uORF**	*OsNLA1* (Os07g0673200)	upstream	confers phosphate-dependent induction	Rice	[126]
**IR**	*ATGGT-IB* (At2g39550)	downstream	enhanced splicing upon cold	*Arabidopsis*	[119]
	n.a. (At2g43160)	downstream	enhanced splicing upon cold	*Arabidopsis*	[119]
	n.a. (At3g47630)	downstream	enhanced splicing upon cold	*Arabidopsis*	[119]
	*CDKG1* (At5g63370)	downstream	altered subcellular localization	*Arabidopsis*	[133]
	n.a. (At3g48070)	downstream	target of the LEF	*Arabidopsis*	[129]
	n.a. (At2g37510)	downstream	target of the LEF	*Arabidopsis*	[129]
	n.a. (At3g19920)	downstream	target of the LEF	*Arabidopsis*	[129]
	n.a. (At4g30993)	downstream	target of SME1	*Arabidopsis*	[120]
	*RD28/PIP2C* (At2g37180)	downstream	target of SME1	*Arabidopsis*	[120]
	n.a. (At3g26360)	downstream	target of SME1	*Arabidopsis*	[120]
	*TTA2/OBE4* (At3g63500)	downstream	target of SME1	*Arabidopsis*	[120]
	*IPGAM2* (At3g08590)	downstream	target of SME1	*Arabidopsis*	[120]
	*SPL2* (At5g43270)	downstream	target of SE	*Arabidopsis*	[53]
	*APE2* (At5g46110)	downstream	target of SE	*Arabidopsis*	[53]
	*HsfA2* (At2g26150)	downstream	target of SE	*Arabidopsis*	[53]
	*SnRK2.8* (At1g78290)	downstream	target of SE	*Arabidopsis*	[53]
	*YUC3* (At1g04610)	downstream	target of CUV	*Arabidopsis*	[127]
	*SHY2/IAA3* (At1g04240)	downstream	target of CUV	*Arabidopsis*	[127]
	*AXR3/IAA17* (At1g04250)	downstream	target of CUV	*Arabidopsis*	[127]
	*NPH4/ARF7* (At5g20730)	downstream	target of CUV	*Arabidopsis*	[127]
	*SLR/IAA14* (At4g14550)	downstream	target of CUV	*Arabidopsis*	[127]
	n.a. (At4G27050)	downstream	target of AtNSRa/b	*Arabidopsis*	[54]
	*AtDRM2, DAP2* (At2G33830)	downstream	target of AtNSRa/b	*Arabidopsis*	[54]

* non-annotated (n.a.) Abbreviations: *HID1*, HIDDEN TREASURE 1; *LEF*, LEFKOTHEA; *SME1,* Sm protein E1; *SE*, SERRATE; *CUV*, CLUMSY VEIN; *AtNSR1a/b*, NUCLEAR SPECKLE RNA-BINDING PROTEIN a/b.
